# Einschätzung von kariösen Zahnläsionen und Therapieoptionen: Vergleich der Kenntnisse von Studierenden der Zahnmedizin und AssistenzzahnärztInnen für Kieferorthopädie

**DOI:** 10.1007/s00103-023-03799-w

**Published:** 2023-11-09

**Authors:** Anahita Jablonski-Momeni, Heike Korbmacher-Steiner

**Affiliations:** Medizinisches Zentrum für Zahn‑, Mund- und Kieferheilkunde, Abteilung für Kieferorthopädie, Philipps-Universität Marburg, Georg-Voigt-Str. 3, 35039 Marburg, Deutschland

**Keywords:** Karies, Kariesdetektion, Kariesrisikoerfassung, Kariesmanagement, Zahnmedizinstudierende, ICDAS, ICCMS, Caries, Caries detection, Caries risk assessment, Caries management, Dental students, ICDAS, ICCMS

## Abstract

Um das Zahnmedizinstudium zu optimieren, sollten etablierte Verfahren zur Kariesdiagnostik und -therapie wie das „International Caries Classification and Management System“ (ICCMS) in den Lehrplan integriert werden. Vorbereitend wurde der Kenntnisstand von Zahnmedizinstudierenden zu Kariesdetektion und -management erhoben, um Defizite in der Lehre gezielt aufgreifen zu können. Ergänzend wurden die erhobenen Daten dem Wissen von klinisch erfahreneren ZahnärztInnen gegenübergestellt. Die Ergebnisse werden in dem vorliegenden Bericht dargestellt und mögliche Konsequenzen für die zahnmedizinische Lehre diskutiert.

26 Studierende im 6. Fachsemester Zahnmedizin und 6 AssistenzzahnärztInnen der Poliklinik für Kieferorthopädie (Philipps-Universität Marburg) standen im Wintersemester 2018/2019 für die schriftliche Befragung zur Verfügung. Für 12 anonymisierte kieferorthopädische Patientenfälle sollten für je einen definierten Zahn der klinische Befund, das Kariesrisiko und die adäquate Behandlung ermittelt werden. Als Referenzwert diente die Konsensentscheidung von 2 erfahrenen ZahnärztInnen. Im Ergebnis lag die Übereinstimmung mit der Referenz bei den Studierenden zwischen 40,7 % und 51,3 % und bei den AssistenzzahnärztInnen zwischen 56,9 % und 75,0 %. Die Kariesausdehnung und die darauf basierende notwendige Behandlung wurden meist unterschätzt.

Neben theoretischen Kenntnissen ist die klinische Erfahrung eine weitere Voraussetzung für eine adäquate Diagnose von Karies und deren Management. Das Unterschätzen von Kariesausdehnung und notwendiger Behandlung sollte bei der Übermittlung von Lehrinhalten berücksichtigt werden. Verfahren wie das ICCMS sollten bereits zu einem frühen Zeitpunkt in das Curriculum integriert werden, damit auch kieferorthopädische PatientInnen effektiv versorgt werden können.

## Abstract (en)

In order to optimize dental education, established procedures for caries diagnosis and therapy, such as the International Caries Classification and Management System (ICCMS), should be integrated into the curriculum. In preparation, the level of knowledge of dental students on caries detection and management was surveyed in order to specifically address deficits in dental teaching. In addition, the data were compared with the knowledge of clinically experienced dentists. The results are presented in this report and possible consequences for dental teaching are discussed.

Twenty-six sixth semester dental students and six assistant dentists at the Department of Orthodontics (Philipps University of Marburg, Germany) were available for the written survey during the winter semester 2018/2019. For 12 anonymized orthodontic patient cases, the clinical findings, caries risk, and adequate treatment were to be determined for each defined tooth. The consensus decision of two experienced dentists served as the reference value. As a result, agreement with the reference ranged from 40.7% to 51.3% for students and from 56.9% to 75.0% for assistant dentists. The extent of caries and the resulting necessary treatment were mostly underestimated.

In addition to theoretical knowledge, clinical experience is another prerequisite for adequate caries diagnosis and its management. The underestimation of caries extent and necessary treatment should be taken into account when teaching content. Procedures such as the ICCMS should be integrated into the dental curriculum at an early stage, so that orthodontic patients can also receive effective care.

## Hintergrund

Die Prävalenz der unbehandelten Karies permanenter Zähne liegt in der Rangfolge der 10 häufigsten Erkrankungen auf Rang 1 (global) bzw. 2 (europäische Union; [1]). Betrachtet man die Mundgesundheitsziele für Deutschland, sollen bis zum Jahr 2030 die permanenten Dentitionen bei 12-Jährigen zu mindestens 90 % kariesfrei sein und der DMF‑T^1^ bei 0,5 gehalten werden [2].

Während einer kieferorthopädischen Therapie mit festsitzenden Apparaturen können, u. a. aufgrund der erschwerten Mundhygiene, vermehrt initiale kariöse Läsionen an den Zähnen auftreten [3]. Ohne adäquate Diagnostik und individuelle Kariesrisikoeinschätzung schreiten unbehandelte initialkariöse Läsionen fort, sodass eine invasive Therapie (z. B. restaurative Versorgung) erforderlich wird.

In der Regel werden kariöse Läsionen zunächst visuell beurteilt, ergänzend können Röntgenbilder zur weiterführenden Diagnostik eingesetzt werden. Das international anerkannte Kariesdiagnoseverfahren ICDAS (International Caries Detection and Assessment System) wurde im Jahr 2002 entwickelt und ermöglicht eine standardisierte visuelle Beurteilung der Karies bereits auf dem Niveau von initialen Schmelzveränderungen. Dadurch wird die frühzeitige Detektion der Karies unterstützt. Entsprechend kann das daraus resultierende Kariesmanagement im frühen Stadium der Kariesentstehung geplant und durchgeführt werden. In der modernen Zahnheilkunde können durch verbesserte präventive und minimal-invasive Maßnahmen zahnsubstanzschonende Behandlungsoptionen eingesetzt werden, sodass eine Progression der Karies verhindert bzw. minimiert werden kann. Hierzu bedarf es jedoch einer Beurteilung des individuellen Kariesrisikos von PatientInnen, d. h. der Einschätzung der Wahrscheinlichkeit, inwieweit der/die PatientIn in Zukunft kariöse Läsionen entwickeln wird. Hierfür wurde als konsequente Weiterführung des ICDAS ein standardisiertes Verfahren zur Erfassung des Kariesrisikos kontinuierlich entwickelt und erstmalig im Jahr 2014 als ICCMS (International Caries Classification and Management System) vorgestellt [4]. Dabei werden patientenbezogene Parameter zusammen mit den Befunden zur Ausdehnung und Aktivität der Karies zusammengeführt, um eine individuelle Behandlung durchführen zu können.

Im Einzelnen besteht das ICCMS aus 4 Elementen, die ein individualisiertes und zahnerhaltendes Kariesmanagement ermöglichen [4]:

- Element 1 – Anamneseerhebung und Erfassung der patientenbezogenen Kariesrisikofaktoren,
- Element 2 – Klassifikation der Karies und Beurteilung der Läsionsaktivität sowie intraorale Kariesrisikobestimmung,
- Element 3 – Entscheidungsfindung: Synthese und Diagnose,
- Element 4 – Kariesmanagement: individualisierte Kariesprophylaxe, (Verlaufs‑)Kontrolle und zahnerhaltende Versorgung.

Zur Vermeidung der Entstehung von initialen Läsionen (im Sinne der Primärprävention), aber auch im Rahmen der Sekundär- und Tertiärprävention gehört die Einschätzung des individuellen Kariesrisikos zur medizinischen Indikationsstellung einer kieferorthopädischen Behandlung bedingungslos dazu. Hierbei kann die Integration des standardisierten „International Caries Classification and Management System“ (ICCMS) erwogen werden [4, 5].

Im Rahmen der zahnmedizinischen Lehre stellen Kariesdetektion und Beurteilung des Kariesrisikos von PatientInnen grundlegende zahnärztliche Säulen dar, da diese später einen wesentlichen Bestandteil der alltäglichen zahnärztlichen Routine beinhalten. Das standardisierte und international akzeptierte visuelle Verfahren der Kariesdetektion ICDAS wurde bereits erfolgreich in Lehre und Forschung etabliert [6, 7]. Als Konsequenz einer detaillierten Detektion der Karies ist die Beurteilung des Kariesrisikos von großer klinischer Bedeutung, da darauf basierend individuelle Therapiemaßnahmen insbesondere im Kontext der Prävention bzw. minimal-invasiver Methoden eingeleitet werden. Hierbei kann das ICCMS-Konzept herangezogen werden. Daher sollten solche weiterführenden Ansätze ebenfalls in der regulären zahnmedizinischen Lehre abgebildet sein. Die Relevanz des Themas ergibt sich neben der konsequenten Fortführung des ICDAS auch aus der Integration des „Nationalen Kompetenzbasierten Lernzielkatalogs Zahnmedizin“ (NKLZ) in der Lehre. Im derzeit vorliegenden NKLZ ist das Kariesmanagement enthalten und sollte idealerweise auch in die überarbeitete Version NKLZ 2.0 übernommen werden.

Um Aspekte wie Detektion und Management von kariösen Veränderungen bei kieferorthopädischen PatientInnen noch besser in die Lehre integrieren zu können, ist es zunächst wichtig, das vorhandene Wissen von Studierenden zu evaluieren und darauf basierend maßgeschneiderte Lehrinhalte zu entwickeln. Als Vorbereitung wurden mittels Fragebögen die Kenntnisse bzw. die Entscheidungskompetenz von Zahnmedizinstudierenden (Undergraduates) erfasst und dem Wissen von AssistenzzahnärztInnen im Fach Kieferorthopädie (Postgraduates) gegenübergestellt. Die Ergebnisse werden im vorliegenden Bericht dargestellt und die möglichen Konsequenzen für die zahnmedizinische Lehre diskutiert.

## Durchführung der Studie

Für die Studie konnten im Wintersemester 2018/2019 freiwillige Studierende des 6. Semesters Zahnmedizin (1. klinisches Semester, im Folgenden Undergraduates) im Zentrum für Zahn‑, Mund- und Kieferheilkunde der Philipps-Universität rekrutiert werden. Als Postgraduates nahmen AssistenzzahnärztInnen der Poliklinik für Kieferorthopädie teil, die sich in der Weiterbildung zum/FachzahnarztIn für Kieferorthopädie befanden und nach dem Staatsexamen mindestens ein Jahr als VorbereitungsassistentIn in zahnärztlichen Praxen tätig waren.

Die Kariesdiagnose gehörte zum Lehrplan des 6. Semesters. Die Studierenden hatten zum Zeitpunkt der Befragungen noch keine klinische Erfahrung, wurden aber bereits vor der Studie in entsprechenden Vorlesungen in das ICCMS eingeführt. Im Einzelnen bestand diese Einführung aus einer 2‑stündigen Vorlesung in Präsenz zu den Kriterien des ICDAS (International Caries Detection and Assessment System; [4]). Es wurden Informationen über den Hintergrund von ICDAS und die Klassifikation mittels Codes vorgestellt und Übungen zu ausgewählten Bildern durchgeführt und diskutiert. Anschließend wurden die Studierenden mit der Internetseite des ICCMS vertraut gemacht. Dort konnten sie nach einer kostenfreien Registrierung auf ein 60-minütiges E‑Learning-Video^2^ zugreifen, in dem die Kariesklassifikation erläutert und anhand von Beispielen geübt werden konnte. Keine/r der Teilnehmenden besaß klinische Erfahrungen mit dem detaillierten Kariesmanagement-System ICCMS.

**Figure 1:**
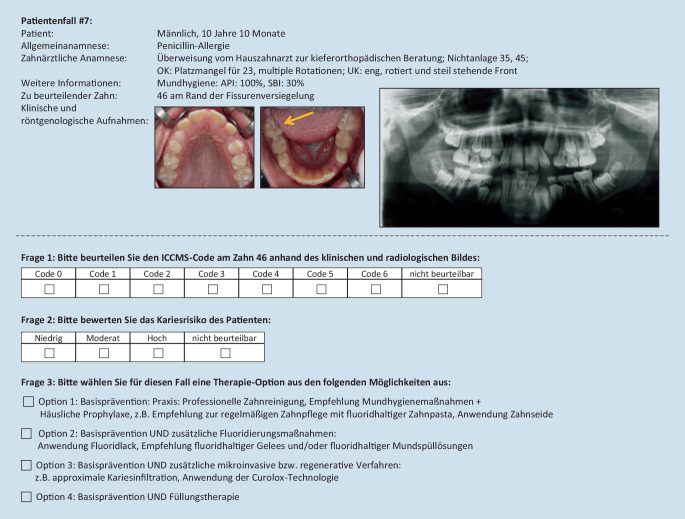

Die schriftlichen Befragungen wurden anhand eines strukturierten Fragebogens mit anonymisierten Patienteninformationen und -bildern durchgeführt. In Anlehnung an die Elemente des ICCMS [4] wurden anonymisierte Patientenfälle (*n* = 12) zusammengestellt (Beispiel Abb. 1). Zu jedem Patientenfall wurden klinische und radiologische Aufnahmen zusammengestellt. Neben Angaben zum Alter (Jahre, Monate) und Geschlecht wurden zusätzliche Informationen angegeben, anhand derer das Kariesrisiko charakterisiert werden konnte. Die Aufgabe bestand darin, für einen definierten Zahn die Ausdehnung der Karies nach den ICCMS-Kriterien wie folgt zu beurteilen:

- Code 0: keine sichtbare Karies,
- Code 1: erste visuelle Änderung im Schmelz,
- Code 2: deutliche visuelle Änderung im Schmelz,
- Code 3: Demineralisation bzw. Verlust der Schmelzstruktur ohne sichtbares Dentin,
- Code 4: Karies im Dentin ohne Oberflächenverlust,
- Code 5: deutliche Kavitätenbildung mit sichtbarem Dentin,
- Code 6: großflächige Kavitätenbildung mit sichtbarem Dentin.

Basierend auf dem Befund und Angaben zum/zur PatientIn sollte eine Einschätzung des Kariesrisikos vorgenommen sowie aus einer vorgegebenen Maßnahmenliste ein Behandlungsvorschlag ausgewählt werden. In Abb. 1 ist exemplarisch ein Fall mit den entsprechenden Fragen und Antwortmöglichkeiten dargestellt. Die Patientenfälle wurden als digitale Dateien zur Verfügung gestellt. Nach 10 Tagen wurden alle Fragebögen ohne Zuordnung von Namen zentral erfasst und ausgewertet. Als Referenzstandard für die Antworten galt die Konsensentscheidung von 2 erfahrenen Zahnärztinnen (Lehrende), die im Vorfeld der Befragungen die Patientenfälle bewertet hatten.

Die statistische Auswertung erfolgte mit dem Statistikprogramm MedCalc for Windows, Version 20.216 (MedCalc Software, Ostend, Belgium). Die Übereinstimmung zwischen den Antworten der StudienteilnehmerInnen und dem Referenzstandard wurden prozentual dargestellt und mit Cohens Kappa-Koeffizienten berechnet.

## Ergebnisse der Studie

Insgesamt nahmen 26 Studierende (von 27 im Semester) sowie 6 AssistenzzahnärztInnen aus der Poliklinik für Kieferorthopädie an der Studie teil.

**Figure 2:**
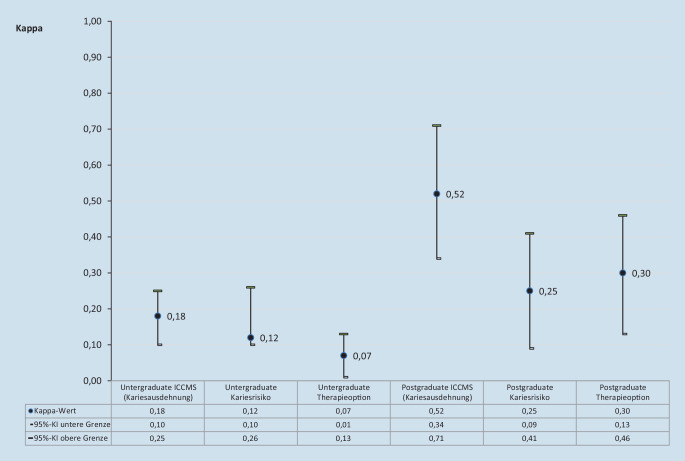

Die Kappa-Werte für die einzelnen Variablen sind in Abb. 2 dargestellt. Für Undergraduates entsprachen alle Kappa-Werte (0,07–0,18) einer geringen Übereinstimmung zur Referenz [8]. Die Kappa-Werte für die Postgraduates lagen zwischen 0,25 und 0,52 und somit im Bereich einer ausreichenden bis moderaten Übereinstimmung.

**Figure 3:**
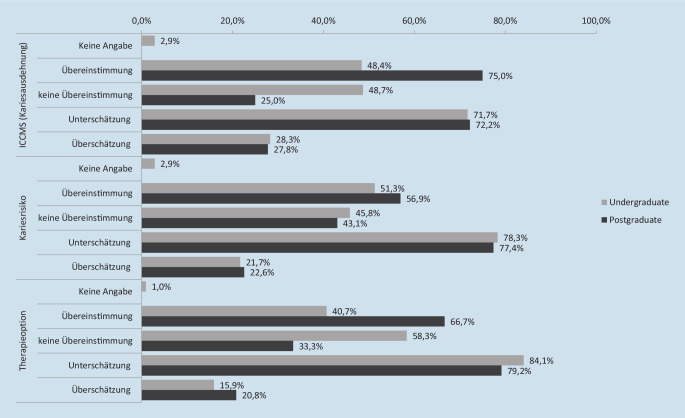

Die Übereinstimmung der Antworten zum Referenzstandard sowie der Anteil von Unter- und Überschätzung sind in Abb. 3 dargestellt. Die Übereinstimmung der Angaben zur Referenz lag für Studierende zwischen 40,7 % und 51,3 % und für AssistenzzahnärztInnen zwischen 56,9 % und 75,0 %. Alle TeilnehmerInnen zeigten eine Tendenz zur Unterschätzung der Schweregrade (ICCMS, Kariesrisiko) sowie zur ausgewählten Therapieoption.

## Diskussion

Orale Erkrankungen stellen ein signifikantes globales Gesundheitsproblem dar [1]. Daher benötigt das Thema Mundgesundheit und insbesondere die Prävention von Karies und Parodontopathien besondere Aufmerksamkeit bereits in der Lehre. Dies ist bereits im Nationalen Kompetenzbasierten Lernzielkatalog Zahnmedizin definiert. Mit der Ausgestaltung der neuen Zahnärztlichen Approbationsordnung (ZApprO^3^), die seit dem Wintersemester 2021/22 gilt, wurden Inhalte des Studiums neu organisiert. Bereits im zweiten Abschnitt der zahnärztlichen Prüfung sollen Studierende fächerübergreifend zeigen, dass sie in der Lage sind, klinisch-zahnmedizinische Zusammenhänge zu erfassen und die für die Fortsetzung des klinischen Studiums und der damit verbundenen Ausbildung am/an PatientInnen notwendigen Kenntnisse und Fähigkeiten zu besitzen. Zu diesen Inhalten gehören u. a. die richtige Diagnose und das Management der Karies in allen Ausprägungen. Weiterhin wurden Unterrichtsveranstaltungen im Querschnittsbereich Gesundheitswissenschaften (u. a. mit den Schwerpunkten Epidemiologie und Prävention) verpflichtend eingeführt. Hierzu gehören synoptische Ansätze in der Prävention, Risikoerfassung und Versorgungsplanung, die idealerweise in vorklinische und klinische Kurse der zahnmedizinischen Lehre integriert werden sollten. Weitere Unterrichtsveranstaltungen, für die eine regelmäßige und erfolgreiche Teilnahme bei dem Antrag auf Zulassung zum Dritten Abschnitt der zahnärztlichen Prüfung nachzuweisen ist, sind Praktika der zahnmedizinischen Diagnostik und Behandlungsplanung sowie der kieferorthopädischen Diagnostik und Therapie. Maßnahmen zur Detektion und zum Management von kariösen Läsionen und weiteren Demineralisationen können hier ideal verknüpft werden.

Als Vorbereitung für die Integration solcher Lehrinhalte wurden Kenntnisse bzw. die Entscheidungskompetenz von Zahnmedizinstudierenden sowie bereits approbierten ZahnärztInnen hinsichtlich Erkennung und Management von kariösen Läsionen mittels eines Fragebogens erfasst. Erhoben wurden die Einschätzungen von Studierenden im 6. Fachsemester Zahnmedizin (ohne klinische Erfahrung) und AssistenzzahnärztInnen für Kieferorthopädie, die erste klinische Erfahrungen in der Kariesdiagnose vorweisen konnten.

Die Notwendigkeit einer systematischen und fundierten Ausbildung von Zahnmedizinstudierenden in der Kariologie ist seit Langem anerkannt [9]. Während die Kariesdetektion mit standardisierten Systemen wie ICDAS im Grundstudium Zahnmedizin inzwischen etabliert ist [6, 7], sind Elemente zur Bewertung von Kariesrisiko und -management nicht generalisiert Gegenstand eines systematischen Ansatzes. Um eine Verlagerung zu einer stärker präventiven und patientenorientierten Behandlungsphilosophie zu unterstützen, wurde das ICCMS als Rahmenwerk entwickelt, das Daten zur Risikobewertung mit Kariesklassifizierungen zusammenführt [4]. Das Ziel war, KlinikerInnen und Lehrende systematisch dabei zu unterstützen, PatientInnen mit erhöhtem Kariesrisiko zu identifizieren und die Zähne, wo immer möglich, non- bzw. minimal-invasiv zu versorgen. Im ICCMS-Konzept ist die Behandlung der Läsionen an die Diagnose der einzelnen Läsionen gebunden. So werden beispielsweise erste aktive Läsionen im Allgemeinen nicht operativ behandelt, während moderate bzw. extensive Läsionen meist restaurativ und nach bester Möglichkeit zahnsubstanzerhaltend behandelt werden. Grundlage hierfür ist der individuelle Risikomanagementplan, der auch Recall-Intervalle und die Überprüfung der Verhaltensänderung des/der PatientIn umfasst.

Für den hier angewendeten Fragebogen wurden die Patientenfälle und die dazugehörigen Fragen an die ICCMS-Leitlinien angepasst, wobei diese 4 Schlüsselelemente berücksichtigt wurden: Anamnese, Klassifizierung, Entscheidungsfindung und Management. Alle Informationen wurden vereinfacht und prägnant dargestellt, da die Teilnehmenden keine (Studierende im 6. Semester) bzw. geringe Erfahrungen (AssistenzzahnärztInnen) in der klinischen Zahnmedizin hatten. Die gesammelten Informationen zeigten, dass die Undergraduates nur geringe Übereinstimmungen mit den Referenzuntersuchern zeigten, während die Postgraduates höherer Übereinstimmungswerte erzielten (Abb. 2). Die prozentuale Übereinstimmung mit den Referenzwerten betrug bei den Studierenden im besten Fall die Hälfte (Kariesrisiko, 51,3 %), hingegen zeigten Postgraduates mit 1–2 Jahren klinischer Erfahrung bis zu 75 % Übereinstimmung (Kariesausdehnung nach ICCMS). Interessanterweise zeigten die teilnehmenden Postgraduates trotz der deutlich besseren Übereinstimmungen mit der Referenz auch eine Tendenz zur Unterschätzung sowohl der Läsionsausdehnung und in der Konsequenz des Kariesrisikos und der Versorgungsform.

Der hier vorgestellte Fragebogen wurde auch in einer Studie an einem weiteren Standort eingesetzt [10]. Dort wurden Studierende des 7. Semesters befragt, die bereits erste klinische Erfahrungen hatten. Sie zeigten im Vergleich zu unseren Studierenden bessere Übereinstimmungen in der Entscheidung über Diagnose und Management von kariösen Läsionen. Die Lehrinhalte zur Kariesdiagnose waren in beiden Zentren nahezu identisch, da beide DozentInnen diese zuvor verglichen und aufeinander abgestimmt hatten. Die fortgeschrittenen Studierenden hatten den theoretischen Kurs bereits in ihrem vorangegangenen Semester besucht. Als die Studie durchgeführt wurde, absolvierten sie bereits den ersten klinischen Kurs in der präventiven und konservierenden Zahnheilkunde. Dennoch wurde die Tendenz zur Unterschätzung der Befunde und des nötigen Kariesmanagements auch bei den Studierenden im fortgeschrittenen Semester beobachtet. Möglicherweise sind Studierende mit wenig klinischer Erfahrung eher in Sorge, kariöse Läsionen zu überschätzen, da hier invasivere Versorgungsmaßnahmen notwendig wären. Andere Studien haben gezeigt, dass Zahnmedizinstudierende im letzten Studienjahr dazu neigen, invasivere Behandlungsoptionen zu wählen, mit einer Tendenz zur Überbehandlung [11, 12]. Es ist davon auszugehen, dass erfahrenere Kliniker ihre Entscheidungen auf frühere Behandlungserfahrungen stützen, während diejenigen mit geringerer klinischer Erfahrung bzw. Studierende eher noch Lehrbücher und Leitfäden als Entscheidungshilfen für Diagnose und Behandlungsplanung verwenden würden [13, 14].

Obwohl die Bewertung der Aktivität einer Läsion ein wichtiger Teil des Prozesses der Kariesdiagnose ist, wurde sie in unserem Fragebogen nicht berücksichtigt. Dies lag vor allem daran, dass die Beurteilung der Kariesaktivität in der Regel auf mehreren klinischen Variablen beruht (u. a. Vorhandensein von Plaque, taktilen Merkmalen wie der Oberflächenrauheit oder der Blutungsneigung der Gingiva) und normalerweise kein einzelner Faktor für die Definition der Kariesaktivität herangezogen werden kann [15]. In unseren Falldarstellungen konnten solche Variablen nicht in geeigneter Weise dargestellt werden. Diese fehlenden Informationen könnten dazu geführt haben, dass die Notwendigkeit einer Behandlung unterschätzt wurde. Nichtsdestotrotz muss der Ausbildung von Studierenden in der Aktivitätsbewertung in einem klinischen Umfeld große Bedeutung beigemessen werden.

Es kann argumentiert werden, dass es sich hier um eine geringe Anzahl von Teilnehmenden handelt und die Ergebnisse daher nicht generalisierbar sind. Die Gruppengröße ergab sich aus der Anzahl der verfügbaren Teilnehmenden im Semester sowie in der Abteilung. Um die kleine Fallzahl zu berücksichtigen, wurden die Ergebnisse deskriptiv berichtet. Diese sollten zunächst für die eigene Lehrplanung unterstützend herangezogen werden, ohne eine Generalisierung der Aussagen für andere Standorte zu beabsichtigen.

Es wurde bereits gezeigt, dass durch zusätzliche Theorieeinheiten die Reproduzierbarkeit der Antworten von Studierenden, die gerade mit dem Erlernen der ICDAS-Methode begonnen hatten, verbessert werden konnte [6]. Andere AutorInnen untersuchten die Anwendung des ICDAS in 3 verschiedenen Gruppen und stellten fest, dass die Unterschiede in der Übereinstimmung zwischen Studierenden, Hochschulabsolventen und Lehrenden statistisch nicht signifikant waren [16]. Die Autoren schlussfolgerten, dass frühere klinische Erfahrungen beim Erlernen des ICDAS keine wesentliche Rolle spielten. Andere haben jedoch gezeigt, dass Studierende mit geringerer klinischer Erfahrung im Gegensatz zu Hochschulabsolventen Schwierigkeiten bei der Beurteilung des Kariesmanagements hatten, insbesondere bei initialkariösen Läsionen [17].

Aktuelle repräsentativ erhobene Daten zeigen zwar einen Rückgang der Kariesprävalenz bei Jugendlichen und Erwachsenen in Deutschland [18]. Global betrachtet stellt Karies im permanenten Gebiss aber immer noch eine der häufigsten Erkrankungen dar [1]. Daher ist eine möglichst frühzeitige standardisierte Kariesdiagnose essenzieller Bestandteil einer minimal-invasiven und präventiven Zahnheilkunde. Entsprechend kann das daraus resultierende Kariesmanagement im frühen Stadium der Kariesentstehung geplant und durchgeführt werden. Hier kann als Weiterführung des ICDAS das standardisierte Verfahren zum Kariesmanagement (ICCMS) bereits in der Lehre etabliert werden, bei dem patientenbezogene Parameter zusammen mit den Befunden zur Ausdehnung und Aktivität der Karies in individualisierte und der aktuellen wissenschaftlichen Evidenz angepasste Behandlungsplanung resultieren [4].

## Fazit

Es kann festgestellt werden, dass die Erfassung von kariösen Läsionen, das Einschätzen des Kariesrisikos sowie die adäquate Behandlungsplanung theoretische Kenntnisse und klinische Erfahrung erfordern. Das ICCMS sollte bereits zu einem frühen Zeitpunkt in das zahnmedizinische Curriculum integriert werden, z. B. um die Relevanz der Entstehung von Initialläsionen bei Multibracketapparatur-Patienten frühzeitig darzustellen und entsprechende Versorgungskonzepte zu unterrichten. Die Überlegungen haben mittlerweile zur Einbindung entsprechender Lehrinhalte in der postgradualen Weiterbildung im Rahmen der hessischen Weiterbildung zum/zur FachzahnarztIn für Kieferorthopädie im Rahmen des NEBEOP (Network of Erasmus Based European Orthodontic Postgraduate Programmes) geführt. Aufgrund der Einführung der neuen Approbationsordnung befindet sich die Umsetzung der Lehrinhalte derzeit im Finalisierungsprozess. Die Fähigkeit zur Detektion und Versorgung von kariösen Läsionen dient der Optimierung des synoptisch integrativen Managements der initialen Karies im Sinne einer präventionsorientierten Zahnmedizin. Hierzu sollten die Bereiche Zahnerhaltung, Kinderzahnheilkunde und Kieferorthopädie gemeinsame Konzepte entwickeln und curricular implementieren.

## Footnotes

1 DMF-T-Index: Summe aller kariösen („decayed“), wegen Karies fehlenden („misssed“) und restaurativ behandelten („filled“) Zähne.

2 https://www.iccms-web.com/content/resources/elearning; letzter Zugriff: 17.10.2023.

3 Approbationsordnung für Zahnärzte und Zahnärztinnen (ZApprO): https://www.gesetze-im-internet.de/zappro/BJNR093310019.html.

## References

[1] Benzian H, Listl S (2021). Globale Mundgesundheit im internationalen Gesundheitspolitischen Rampenlicht. Bundesgesundheitsbl.

[2] Ziller S, Jordan AR, Oesterreich D (2021). Mundgesundheitsziele für Deutschland 2030: Karies und Parodontitis weiter reduzieren sowie Prävention verbessern. Bundesgesundheitsbl.

[3] Ren Y, Jongsma MA, Mei L, van der Mei HC, Busscher HJ (2014). Orthodontic treatment with fixed appliances and biofilm formation—a potential public health threat?. Clin Oral Investig.

[4] Pitts NP, Ismail AI, Martignon S, Ekstrand K, Douglas G, Longbottom C (2014) International caries classification and management system (ICCMS). https://www.iccms-web.com/content/resources/iccms-icdas-publications. Zugegriffen: 16. Mai 2023

[5] Pitts NB, Mazvet ME, Mayne C (2018). Shaping the future of dental education: caries as a case-study. Eur J Dent Educ.

[6] Jablonski-Momeni A, Busche J, Struwe C, Lange J, Heinzel-Gutenbrunner M, Frankenberger R, Pieper K (2012). Use of the international caries detection and assessment system two-digit coding method by predoctoral dental students at philipps university of Marburg, Germany. J Dent Edu.

[7] Jablonski-Momeni A, Hoppe R, Stachniss V, Méndez-Vilas A, Díaz J (2014). Use of digitally optimized images of ICDAS caries codes by undergraduate dental students. Microscopy: advances in scientific research and education.

[8] Landis JR, Koch GG (1977). The measurement of observer agreement for categorical data. Biometrics.

[9] Schulte AG, Pitts NB, Huysmans MC, Splieth C, Buchalla W (2011). European core curriculum in cariology for undergraduate dental students. Eur J Dent Educ.

[10] Jablonski-Momeni A, Korbmacher-Steiner A, Temming A, Wernke P, Heinzel-Gutenbrunner M, Haak R, Krause F (2022). Knowledge of undergraduate dental students regarding management of caries lesions. BDJ Open.

[11] Bervian J, Tovo MF, Feldens CA, Brusco LC, Rosa FMD (2009). Evaluation of final-year dental students concerning therapeutic decision making for proximal caries. Braz oral res.

[12] Bussaneli DG, Boldieri T, Diniz MB, Lima Rivera LM, Santos-Pinto L, Cordeiro RDCL (2015). Influence of professional experience on detection and treatment decision of occlusal caries lesions in primary teeth. Int J Paediatr Dent.

[13] Gowda D, Lamster IB (2011). The diagnostic process. Dent Clin North Am.

[14] Baelum V, Hintze H, Wenzel A, Danielsen B, Nyvad B (2012). Implications of caries diagnostic strategies for clinical management decisions. Community Dent Oral Epidemiol.

[15] Ekstrand KR, Zero DT, Martignon S, Pitts NB, Pitts NBH (2009). Lesion activity assessment. Detection, assessment, diagnosis and monitoring of caries. Monographs in oral science.

[16] Zandona AGF, Al-Shiha S, Eggertsson H, Eckert G (2009). Student versus faculty performance using a new visual criteria for the detection of caries on occlusal surfaces: an in vitro examination with histological validation. Oper Dent.

[17] El-Damanhoury HM, Fakhruddin KS, Awad MA (2014). Effectiveness of teaching international caries detection and assessment system II and its e-learning program to freshman dental students on occlusal caries detection. Eur J Dent.

[18] Jordan AR, Micheelis (Hrsg.) (2016) Fünfte Deutsche Mundgesundheitsstudie (DMS V). Institut der Deutschen Zahnärzte, Deutscher Zahnärzte Verlag DÄV: Köln Germany

